# Development and validation of a virtual teaching method for minimally invasive surgery skills: a prospective cohort study

**DOI:** 10.1097/JS9.0000000000002053

**Published:** 2024-08-26

**Authors:** Bibek Das, Frances Ledesma, Ravi Naik, Sarah Law, Payam Soleimani-Nouri, Omar A Khan, George Mylonas, Madhava Pai, Hutan Ashrafian, Duncan Spalding, Matyas Fehervari

**Affiliations:** a Department of Surgery and Cancer, Faculty of Medicine, Imperial College London; b East Suffolk and North Essex NHS Foundation Trust; c St George’s University Hospitals NHS Foundation Trust; d Maidstone and Tunbridge Wells NHS Trust, UK

**Keywords:** laparoscopic skills, medical education, minimally invasive surgery, online teaching

## Abstract

**Introduction:**

The COVID-19 pandemic led to a significant reduction in operative exposure for surgical trainees, necessitating alternative training methods to mitigate the impact on surgical education. This study sought to evaluate whether minimally invasive surgery (MIS) skills could be taught remotely using widely available technology with objective assessments of proficiency.

**Methods:**

This was a pilot observational study with a comparative assessment of face-to-face (F2F) and virtual training of novice learners in MIS skills. Performance and objective cognitive workload parameters [Surgical Task Load Index (SURG-TLX) score, heart rate, and pupil metrics] were evaluated. The assessments were peg transfer [McGill Inanimate System for Training and Evaluation of Laparoscopic Skills (MISTELS)] and suturing [Suturing Training and Testing (SUTT)] tasks performed using box trainers. Virtual teaching was conducted by expert trainers using a web-based streaming platform.

**Results:**

Technical challenges of delivering a virtual MIS skills course were addressed after a pilot course. Participants (*n*=20) in the final course had similar baseline characteristics and were randomly allocated to F2F (*n*=8) and virtual (*n*=12) teaching groups. Participants in the online group completed the peg transfer task faster than the F2F group (11.25 min vs. 16.88 min; *P*=0.015). There were no significant differences in all other MISTELS and SUTT performance measures between groups. Cognitive workload parameters (SURG-TLX score, heart rate, and pupil metrics) were also similar between groups.

**Conclusion:**

This study has demonstrated that virtual teaching of MIS skills using a web-based streaming platform is feasible and effective, providing the foundation for low-cost, effective, and scalable MIS skills programs in the future.

## Introduction

Highlights

- Evidence for the effectiveness of virtual vs face-to-face teaching is needed.
- A pragmatic and scalable minimally invasive surgery skills course was developed.
- Performance metrics and cognitive workload parameters were used for assessment.
- Equivalent workload and skills acquisition were found in both formats.

At a time of reduced hands-on training in surgical skills, there has been greater reliance on simulation courses to teach basic surgical skills to early years surgical trainees^1,2^. The COVID-19 pandemic, in particular, led to a significant reduction in operative exposure for surgical trainees, necessitating alternative training methods to mitigate the impact on surgical education^3^. Surgical skills courses, however, can be difficult and expensive to deliver face-to-face (F2F). Virtual teaching could address these challenges and improve accessibility for surgical trainees, reducing the cost, time, and carbon footprint from travel. Online teaching could also be extended beyond training novice learners and allow skilled surgeons at remote sites to learn new surgical skills through mentorship from expert trainers based in ‘hub’ centers (tele-mentoring)^4^. The 2030 Lancet Commission on Global Surgery underscored the importance of providing safe and efficient surgical training in low-income nations and middle-income nations to build capacity and encourage retention of skilled staff in communities most in need^5^. Tele-mentoring helps overcome numerous logistical challenges related to distance, time, and the costs involved when volunteer educators travel to low resourced countries to conduct training. In these settings, tele-mentoring thus offers the critical advantage of making surgical expertise available to patients needing procedures in regions that might lack specialized care.

Before tele-education methods can be adopted widely, a thorough evaluation of quality and validation against traditional F2F teaching methods is required^6^. We have previously demonstrated that basic open surgical skills (suturing, vascular anastomosis, and tendon repair) can be taught remotely and can achieve equivalent competence to traditional F2F teaching^7^. Both large-group didactic teaching in a virtual ‘main stage’ and small-group teaching in virtual ‘breakout rooms’ were delivered using an online streaming platform. This enabled 553 delegates from 20 different countries to attend and receive training in basic surgical skills. The postcourse evaluation demonstrated that competency, satisfaction, and teaching quality were equivalent to traditional F2F teaching. This raised the question of whether more advanced surgical skills could be taught successfully in a virtual format.

Teaching minimally invasive surgical (MIS) skills such as intracorporeal suturing remotely presents additional challenges compared to teaching open surgical skills. MIS is inherently more difficult to learn than open surgery due to the loss of depth perception, limited range of movement, and inversion of movement (fulcrum effect)^8^. Closed-box trainers are now widely available and have been shown to faithfully recapitulate these MIS challenges^8^. Demonstrating MIS skills, providing feedback, and correcting mistakes during training sessions easily are notable advantages of F2F teaching. Previous attempts at remote teaching have involved at-home self-directed skills training with video assessment and virtual reality (VR) based tele-mentoring^9,10^. Self-directed assessment lacks one-to-one mentorship from expert surgeons, whereas VR-based techniques, which require specialized headsets, are not yet widely available. These studies have also conducted assessments with simple performance metrics only, without an evaluation of nontechnical skills (e.g. situational awareness), which can be impaired by excessive cognitive workload.

The importance of cognitive workload in medicine and surgery is increasingly being recognized as a construct that affects both technical and nontechnical skills^11^. Validated measures of cognitive workload measurement exist; however, these are at risk of subjective bias. Objective measures, such as the use of sensors, aim to address this and have been used across various specialities and surgical approaches^12^. A single approach to measure cognitive workload, however, has yet to be identified, although the use of multimodal frameworks has shown some promise to tackle this^13^. Since cognitive load decreases with increasing proficiency, its assessment using physiological parameters may help develop more effective training methods^14^.

The aim of this study was to develop and evaluate a virtual teaching course of MIS skills to novice learners using widely available technology. Since performance-based metrics alone may be insufficient to reveal trainees’ actual state of readiness, we also incorporated subjective and objective measures of cognitive load. We hypothesized that the method would provide equivalent competency, but would be more economical, accessible, and sustainable compared with standard F2F teaching in terms of performance and cognitive load.

## Methods

Two 1-day courses were conducted in the Clinical Skills Unit at St Mary’s Hospital, London, UK on 11th March 2022 and 8th April 2022. The first course was a pilot of the virtual teaching course, and the second course was used for a comparative assessment between virtual and F2F teaching. Challenges with virtual teaching identified in the pilot course were addressed in preparation for the second course. Participants were recruited by national advert and were asked to complete a precourse questionnaire to determine baseline skill levels. Local ethical approval and informed consent were obtained (Imperial College Research Ethics Committee No: 20IC6361). This prospective cohort study has been reported in line with the ‘Strengthening the Reporting of cohort, cross-sectional, and case–control studies in Surgery’ (STROCSS) 2021 criteria^15^ (completed checklist in Supplementary Table 1, Supplemental Digital Content 1, http://links.lww.com/JS9/D364). This study protocol has also been registered with ClinicalTrials.gov (ID: NCT06355596).

### Design

Training and assessments were performed using laparoscopic box trainers and stack systems (Karl Storz). A 10 mm 0° telescope was introduced through a midline port in the box and fixed at a distance that allowed visualization of the entire operating field. Lateral ports were placed for needle holders with a 60° manipulation angle and 2-0 polyglactin 910 sutures were used throughout. Participants underwent training and evaluation using two validated laparoscopic assessments: 1) The peg transfer task from the McGill Inanimate System for Training and Evaluation of Laparoscopic Skills (MISTELS) and 2) the European Academy laparoscopic ‘Suturing Training and Testing’ (SUTT) assessment^16,17^.

The MISTELS peg transfer task involves two pegboards and six pegs. The objective is firstly, to lift each peg from one pegboard with a grasper in the left hand, transfer it to a grasper in the right hand, and then place it on the other pegboard. Once all pegs have been transferred, each peg is then lifted with a grasper in the right hand, transferred to the grasper in the left hand, and placed back on the original pegboard. If a peg is dropped outside the field of view, the peg is discarded. The task is complete when all pegs have been transferred.

The SUTT assessment consists of suturing on a 6 cm diameter white sponge with two rows of four black dots. Participants are asked to complete four intracorporeal knots with the needle entering and exiting through the center of parallel black dots. The intracorporeal knot is formed by a double counter-clockwise knot, followed by a single clockwise knot, and, finally, by a single counter-clockwise knot.

Participants were allocated randomly to two separate rooms for either face-to-face (F2F) teaching or virtual/online teaching by two expert trainers (attending surgeons) per room. All trainers had been fully briefed on the tasks prior to the course. Two facilitators were also allocated to each room and were responsible for setting up the tasks and timekeeping only. For each task, participants watched a video demonstration (5 min), had a practice session with either F2F or virtual trainers (30 min per participant) before a formal assessment (30 min per participant). Videos of the final assessments were recorded using the stack system.

For the F2F training session, trainers were free to interact with all participants, answer questions, and demonstrate the technique on the box trainer if needed. For the virtual training session, trainers based at a nearby hospital and participants used laptops with webcams to communicate. Both trainers and trainees were logged into the MedAll platform (http://www.medall.org), consisting of a ‘main stage’ broadcast onto a large monitor visible to all participants and ‘breakout rooms’ corresponding to webcam broadcasts of monitors from individual stack systems. Participants and facilitators used two-way lightweight Bluetooth headphones to communicate with the trainers. Trainers were able to use the main stage to re-play the demonstration video and highlight key steps, and/or move between breakout rooms to give individual advice and feedback.

### Outcomes

For the MISTELS peg transfer task, time to completion and number of pegs dropped were recorded, in addition to objective and subjective measurements of cognitive load. The surgical task load index (SURG-TLX) is a validated subjective instrument used to measure the overall perceived workload of surgeons during a task^18^. This involves a two-part questionnaire evaluation resulting in a workload score ranging from 0 to 100. Heart rate, pupil size, fixation, and blink frequency were selected as objective parameters of workload. Pupil metrics offer an objective source of mental workload estimation and have been used extensively in the real and simulated surgical settings to demonstrate cognitive workload levels as well as to determine levels of expertise^19–24^. Participants wore heart rate sensors (Consensys Shimmer 3, Shimmer Sensing Inc.) and Pupil Labs Pupil Core eye trackers (Pupil Labs). Both were applied to subjects and signals were confirmed prior to the start of the task. Baseline heart parameters were also recorded, and the eye trackers were calibrated.

For the SUTT assessment, five parameters were measured: the time needed to complete the exercise, the number of mistakes (thread passing out of the dot), the number of traumas (tear made to the tissue), the correct tying of the knot, and good tissue approximation.

Each participant was scored from video recordings of their assessments by Consultant Surgeons blinded to the method of teaching. Baseline characteristics and outcome variables were compared using Welch’s *T*-test or Mann–Whitney *U* test for continuous variables as appropriate, and Fisher exact test for categorical variables. Data were presented as means with SD for continuous variables and frequency with percentage for categorical variables. All statistical analyses were performed using R (version 4.3.0).

## Results

**Figure 1:**
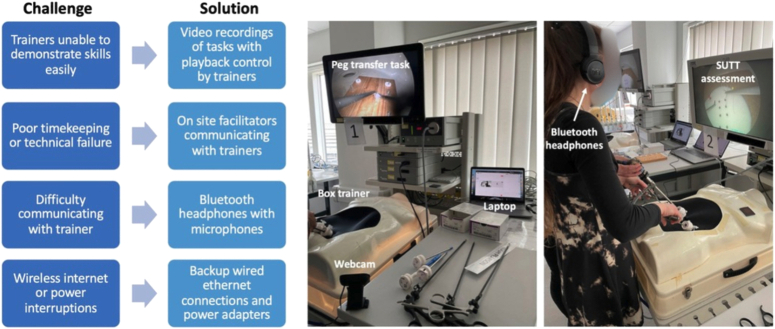
Flowchart showing the challenges identified during a pilot virtual minimally invasive surgery skills course and proposed solutions. The images on the right show the final set-up for the peg transfer task.

During the pilot course, several challenges were identified that were mitigated in the final course (Fig. 1). A video demonstration that could be paused and re-played by trainers was used to narrate key steps of the procedure during the training session. The presence of facilitators in the teaching room was a vital addition as they could ensure strict timekeeping and could bring virtual trainers to students in need of assistance. Facilitators could also quickly address technical problems such as low laptop battery life (providing power chargers) and a drop in wireless internet connection (using wired ethernet cables). Finally, the use of Bluetooth headphones with microphones enabled clear communication between trainer and student compared with using speakers alone.

The baseline characteristics of the participants (*n*=20) evaluated in the final course are displayed in Table 1. The majority of candidates were 2–5 years from graduation (*n*= 6, 80%), had assisted in a median of 50 laparoscopic cases (IQR 8.50–60) and performed a median of 8 laparoscopic cases (IQR 0–20). There were no significant baseline differences between F2F and online teaching groups.

**Table 1:** Baseline characteristics of participants.

Group	F2F (*n*=8)	Online (*n*=12)	*p*
Male (%)	4 (50.0)	9 (75.0)	0.503
Years since graduation (%)			0.128
1 year	0 (0.0)	1 (8.3)	
2–5 years	5 (62.5)	11 (91.7)	
5+ years	2 (25.0)	0 (0.0)	
Medical student	1 (12.5)	0 (0.0)	
Right hand dominance (%)	7 (87.5)	10 (83.3)	1
Lap cases as assistant (median [IQR])	50.00 [17.50–72.50]	50.00 [4.00–52.50]	0.506
Lap cases performed (median [IQR])	12.50 [4.50–23.75]	0.50 [0.00–12.50]	0.281

F2F, face to face.

**Figure 2:**
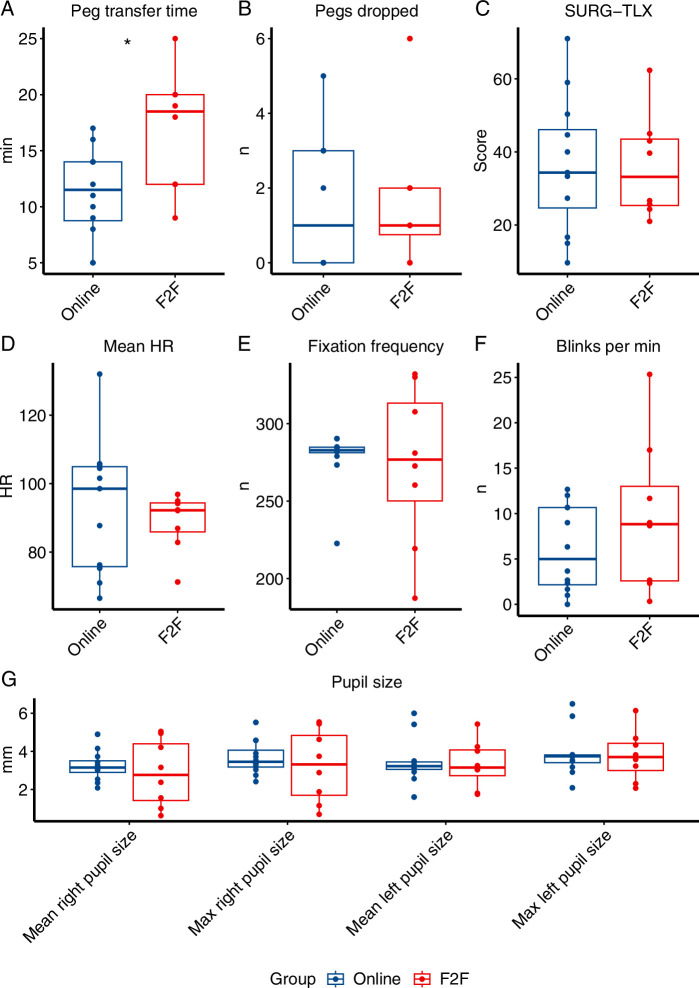
Box and whisker plots showing McGill Inanimate System for Training and Evaluation of Laparoscopic Skills peg transfer task performance and cognitive load metrics comparing online and face-to-face teaching. A, Peg transfer time; B, Pegs dropped; C, Surgical Task Load Index score; D, Mean Heart Rate; E, Fixation frequency; F, Blinks per minute; G, Pupil size.

**Figure 3:**
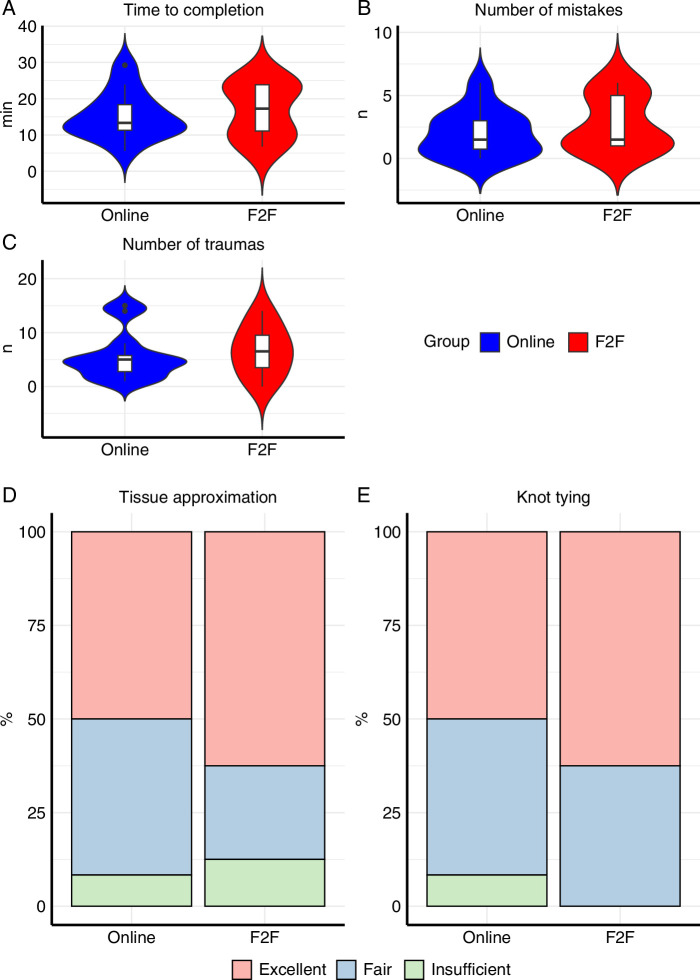
Violin plots and bar charts showing suturing training and testing assessment results comparing online and face-to-face teaching. A, Time to completion; B, Number of mistakes; C, Number of traumas; D, Tissue approximation; E, Knot tying.

The results of the MISTELS peg transfer task are summarized in Figure 2 and Supplementary Table 2 (Supplemental Digital Content 2, http://links.lww.com/JS9/D365). On average, participants in the online training group completed the task faster than the F2F group (11.25 min vs. 16.88 min; *P*=0.015) with no difference in number of pegs dropped (*P*=0.961). All subjective (SURG-TLX score) and objective (heart rate, pupil size, fixation, and blink frequency) parameters of cognitive workload were similar between groups. The results of the SUTT assessment are summarized in Figure 3 and Supplementary Table 3 (Supplemental Digital Content 2, http://links.lww.com/JS9/D365). All evaluated performance measures were similar between groups.

## Discussion

In this study, a pragmatic and scalable virtual teaching format for MIS skills was developed and evaluated in a pilot observational study using traditional and novel objective methods. Important challenges of the virtual format, which impeded communication and learning, were addressed. Delegates who had received virtual teaching achieved similar performance measures in both simple (MISTELS) and advanced (SUTT) MIS skills compared with those taught by in-person trainers. Although participants in the online teaching group completed the MISTELS task faster than the F2F group, larger studies will be needed to confirm whether this is truly due to higher skills acquisition in the online format rather than variability in unreported baseline performance metrics. The important finding is that this virtual teaching format appears to have delivered skills as effectively as F2F with similar performance metrics and cognitive workload, but provided a more cost-effective, accessible, and sustainable option. The use of a low-cost video platform, in particular, is essential to facilitate national/international adoption. By evaluating performance and cognitive load using validated assessments, this data provides ‘relations with other variables’ level of evidence, according to Messick’s validity framework^6^.

Delegates in both F2F and online teaching groups were able to rapidly acquire MIS skills and achieve high scores in the assessments. It has been previously shown that endoscopic suturing skills can be acquired by novice learners in short F2F skills courses irrespective of baseline experience and that these skills can translate to improved performance in the operating room^25,26^. This format could be used for both formative and summative assessments of surgical skills, as part of Objective Structured Assessments of Technical Skills (OSATS)^27^. The MISTELS and SUTT models are objective assessments of laparoscopic skills and were chosen for their simplicity and proven reliability in evaluating core MIS skills^16,17^. Increased stress, which can impair surgical decision-making, is inversely correlated with proficiency and may adversely affect patient outcomes^28^. Thus, the chosen assessment format – evaluating cognitive workload parameters alongside objective performance metrics – enables a holistic overview of technical proficiency and readiness to translate learned skills to clinical practice^12^.

Limitations of this study include the small sample size and lack of replication data, generating high SDs for the measured endpoints. It will be important to confirm that the outcomes of the course can be maintained over successive courses in a larger cohort and that learned skills can be retained. A high-speed internet connection, which is an essential requirement for virtual teaching, may not be available in rural areas of developing countries where remote teaching would be most needed. An additional unanswered question is which types of learners are most suited for the virtual learning format, and perhaps more importantly, which learners would benefit from more F2F training^29^. Future studies could evaluate Kolb's learning styles prior to virtual teaching and identify subgroups who appear to have difficulty acquiring new skills in the virtual format.

In summary, this study shows that a simple virtual teaching format using a web-based streaming platform is feasible and effective for MIS skills teaching. We provide evidence of equivalent cognitive workload and skills acquisition in both virtual and F2F formats. This virtual teaching format could provide the foundation for low-cost, effective, and scalable MIS skills programs in the future.

## Ethical approval

Imperial College Research Ethics Committee; Reference No: 20IC6361.

## Consent

Informed consent was obtained from participants.

## Source of funding

This study was funded by an FST/ASME grant (Project title: ‘Validation of Online Laparoscopic Surgical Training’) awarded to MF (Grant No. FST/ASME/21/009) and partially supported by the EPSRC Transformative Healthcare Technologies grant EP/W004755/1. B.D. is funded by a Medical Research Council fellowship (Grant No. MR/V02955X/1).

## Author contribution

B.D., F.L., R.N., P.S.N., S.L., G.M., and M.P.: contributed to data collection, data analysis, and drafting the paper; M.F., H.A., O.A.K., and D.S.: contributed to conception and design, drafting the paper, critical revision of the paper, and approval of the paper.

## Conflicts of interest disclosure

The authors declare no conflicts of interest.

## Research registration unique identifying number (UIN)

ClinicalTrials.gov ID NCT06355596.

## Guarantor

Matyas Fehervari. E-mail: matyas.fehervari15@imperial.ac.uk.

## Data availability statement

The authors confirm that the data supporting the findings of this study are available within the article [and/or] its supplementary materials.

## Provenance and peer review

Not commissioned, externally peer-reviewed.

## Supplementary Material

SUPPLEMENTARY MATERIAL
