# Supplementary material for: Development and validation of a virtual teaching method for minimally invasive surgery skills: a prospective cohort study
Source: Int J Surg. 2024 Aug 26;110(12):7414–20. doi: 10.1097/JS9.0000000000002053 (PMC11634133; doi:10.1097/JS9.0000000000002053)
Supplement: SUPPLEMENTARY MATERIAL [file js9-110-7414-s002.docx]

Supplementary Table 2. MISTELS peg transfer task with cognitive load measurements.

| Group | F2F (n = 8) | Online (n = 12) | p |
| --- | --- | --- | --- |
| Peg transfer (mean (SD)) |  |  |  |
| Time transferred (min) | 16.88 (5.36) | 11.25 (3.98) | 0.015 |
| Dropped (n) | 1.62 (1.92) | 1.58 (1.78) | 0.961 |
| SURG-TLX score (mean (SD)) | 35.96 (14.10) | 36.31 (18.20) | 0.964 |
| Mean heart rate (mean (SD)) | 88.94 (8.46) | 93.15 (19.70) | 0.58 |
| Mean right pupil size (mm) (mean (SD)) | 2.87 (1.75) | 3.23 (0.77) | 0.527 |
| Max right pupil size (mm) (mean (SD)) | 3.25 (1.90) | 3.66 (0.87) | 0.512 |
| Mean left pupil size (mm) (mean (SD)) | 3.32 (1.24) | 3.45 (1.17) | 0.812 |
| Max left pupil size (mm) (mean (SD)) | 3.77 (1.32) | 3.87 (1.19) | 0.864 |
| Fixation frequency (n) (mean (SD)) | 273.79 (51.23) | 278.28 (18.09) | 0.782 |
| Blinks per minute (n) (mean (SD)) | 9.62 (8.41) | 6.06 (4.70) | 0.238 |

Abbreviations: F2F, face to face; min, minutes; mm, millimetres; SD, standard deviation.

Supplementary Table 3. Suturing Training and Testing Assessment (SUTT)

| Group | F2F (n = 8) | Online (n = 12) | p |
| --- | --- | --- | --- |
| Time to completion (min) (mean (SD)) | 16.66 (7.51) | 15.37 (6.54) | 0.689 |
| Number of mistakes (n) (mean (SD)) | 2.75 (2.19) | 2.00 (1.86) | 0.42 |
| Number of traumas (n) (mean (SD)) | 6.62 (4.66) | 5.67 (4.58) | 0.654 |
| Tissue approximation (n (%)) |  |  | 0.741 |
| Excellent | 5 (62.5) | 6 (50.0) |  |
| Fair | 2 (25.0) | 5 (41.7) |  |
| Insufficient | 1 (12.5) | 1 (8.3) |  |
| Knot tying (n (%)) |  |  | 0.662 |
| Excellent | 5 (62.5) | 6 (50.0) |  |
| Fair | 3 (37.5) | 5 (41.7) |  |
| Insufficient | 0 (0.0) | 1 (8.3) |  |

Abbreviations: F2F, face to face; SD, standard deviation.
